# Epidemiological Characteristics of 2009 (H1N1) Pandemic Influenza Based on Paired Sera from a Longitudinal Community Cohort Study

**DOI:** 10.1371/journal.pmed.1000442

**Published:** 2011-06-21

**Authors:** Steven Riley, Kin O. Kwok, Kendra M. Wu, Danny Y. Ning, Benjamin J. Cowling, Joseph T. Wu, Lai-Ming Ho, Thomas Tsang, Su-Vui Lo, Daniel K. W. Chu, Edward S. K. Ma, J. S. Malik Peiris

**Affiliations:** 1 MRC Centre for Outbreak Analysis and Modelling, Department of Infectious Disease Epidemiology, School of Public Health, Imperial College London, United Kingdom; 2 Department of Community Medicine and School of Public Health, The University of Hong Kong, Hong Kong Special Administrative Region, People's Republic of China; 3 Centre for Health Protection, Department of Health, Government of the Hong Kong Special Administrative Region, People's Republic of China; 4 Hospital Authority, Hong Kong Special Administrative Region, People's Republic of China; 5 Department of Microbiology, Li Ka Shing Faculty of Medicine, The University of Hong Kong, Hong Kong Special Administrative Region, People's Republic of China; 6 HKU-Pasteur Research Center, Hong Kong Special Administrative Region, People's Republic of China; George Washington University, United States of America

## Abstract

**Background:**

While patterns of incidence of clinical influenza have been well described, much uncertainty remains over patterns of incidence of infection. The 2009 pandemic provided both the motivation and opportunity to investigate patterns of mild and asymptomatic infection using serological techniques. However, to date, only broad epidemiological patterns have been defined, based on largely cross-sectional study designs with convenience sampling frameworks.

**Methods and Findings:**

We conducted a paired serological survey of a cohort of households in Hong Kong, recruited using random digit dialing, and gathered data on severe confirmed cases from the public hospital system (>90% inpatient days). Paired sera were obtained from 770 individuals, aged 3 to 103, along with detailed individual-level and household-level risk factors for infection. Also, we extrapolated beyond the period of our study using time series of severe cases and we simulated alternate study designs using epidemiological parameters obtained from our data. Rates of infection during the period of our study decreased substantially with age: for 3–19 years, the attack rate was 39% (31%–49%); 20–39 years, 8.9% (5.3%–14.7%); 40–59 years, 5.3% (3.5%–8.0%); and 60 years or older, 0.77% (0.18%–4.2%). We estimated parameters for a parsimonious model of infection in which a linear age term and the presence of a child in the household were used to predict the log odds of infection. Patterns of symptom reporting suggested that children experienced symptoms more often than adults. The overall rate of confirmed pandemic (H1N1) 2009 influenza (H1N1pdm) deaths was 7.6 (6.2–9.5) per 100,000 infections. However, there was substantial and progressive increase in deaths per 100,000 infections with increasing age from 0.66 (0.65–0.86) for 3–19 years up to 220 (50–4,000) for 60 years and older. Extrapolating beyond the period of our study using rates of severe disease, we estimated that 56% (43%–69%) of 3–19 year olds and 16% (13%–18%) of people overall were infected by the pandemic strain up to the end of January 2010. Using simulation, we found that, during 2009, larger cohorts with shorter follow-up times could have rapidly provided similar data to those presented here.

**Conclusions:**

Should H1N1pdm evolve to be more infectious in older adults, average rates of severe disease per infection could be higher in future waves: measuring such changes in severity requires studies similar to that described here. The benefit of effective vaccination against H1N1pdm infection is likely to be substantial for older individuals. Revised pandemic influenza preparedness plans should include prospective serological cohort studies. Many individuals, of all ages, remained susceptible to H1N1pdm after the main 2009 wave in Hong Kong.

*Please see later in the article for the Editors' Summary*

## Abstract 2

Steven Riley and colleagues analyze a community cohort study from the 2009 (H1N1) influenza pandemic in Hong Kong, and found that more children than adults were infected with H1N1, but children were less likely to progress to severe disease than adults.

## Editors' Summary

**Background:**

From June 2009 to August 2010, the world was officially (according to specific WHO criteria—WHO phase 6 pandemic alert) in the grip of an Influenza A pandemic with a new strain of the H1N1 virus. During this time, more than 214 countries and overseas territories reported laboratory confirmed cases of pandemic influenza H1N1 2009 with almost 20,000 deaths.

While much is already known about patterns of incidence of clinical influenza, the patterns of infection incidence are much more uncertain, because many influenza infections are either asymptomatic or cause only mild symptoms. This means that it is difficult to obtain accurate estimates of risk factors for infection and the overall burden of disease using only clinical surveillance. However, without accurate estimates of infection incidence across different risk groups, it is not possible to establish the number of infections that give rise to severe disease (the per infection rate of severe disease). Consequently, it is difficult to give evidence-based advice for individuals, groups, and populations about the potential benefits of interventions including drugs and vaccines that might reduce the risk of influenza infection.

**Why Was This Study Done?:**

During the 2009 pandemic, some countries and territories, such as Hong Kong, were able to investigate patterns of mild and asymptomatic infection using serological techniques, thus providing information that may help to fill this knowledge gap. Given the high levels of polymerase chain reaction (PCR) testing and the robust reporting of hospital episodes, the main H1N1 pandemic wave in Hong Kong (during September 2009) provided an opportunity to implement a prospective cohort study to investigate the incidence of infection.

**What Did the Researchers Do and Find?** The researchers collected data on the asymptomatic symptoms of influenza by randomly selecting households to participate in the study. Each member of the household willing to participate had a baseline blood sample taken before the main wave of the pandemic (July to September 2009), then, when clinical surveillance suggested that the main peak in transmission had passed, after the main wave (November 2009 to February 2010). During the study period, participants were asked to report any flu-like symptoms in three ways: to phone the study team and report symptoms in real time; to fill out a paper diary with the day and symptoms; and to report symptoms during a follow-up interview. In parallel, the researchers monitored data on every patient with H1N1 admitted to intensive care units or who died while in the hospital. The researchers then estimated the number of H1N1 infections (infection incidence) per severe case by developing a likelihood-based framework. They used a simulation model to investigate alternate study designs and to validate their estimates of the rate of severe disease per infection.

Using these methods, the researchers found that rates of H1N1 infection during the study period decreased substantially with age: for 3–19 years, the attack rate was 39%; 20–39 years, 8.9%; 40–59 years, 5.3%; and 60 years or older, 0.77%. In addition, patterns of symptom reporting indicated that children experienced symptoms more often than adults. The overall rate of confirmed H1N1 deaths was 7.6 per 100,000 infections. However, there was a substantial and progressive increase in deaths per 100,000 infections with increasing age from 0.66 for 3–19 years up to 220 for 60 years and older. Statistical modeling suggested that 56% of 3–19 year olds and 16% of people overall were infected by the pandemic strain up to the end of January 2010.

**What Do These Findings Mean?:**

The results of this study suggest that more children were infected with H1N1 than adults but most of them did not progress to severe disease. Conversely, although fewer older adults were infected with H1N1, this group was much more likely to experience severe disease. Therefore, should H1N1 infection incidence ever increase in older adults, for example by evolving to become more infectious to this group, average rates of severe disease per infection could be much higher than for the 2009 pandemic. Revised pandemic preparedness plans should include prospective serological cohort studies, such as this one, in order to be able to estimate rates of severe disease per infection.

**Additional Information:**

Please access these Web sites via the online version of this summary at http://dx.doi.org/10.1371/journal.pmed.1000442.

- WHO has information about the global response to the 2009 H1N1 pandemic
- WHO also provides recommendations for the H1N1 post-pandemic period
- The government of Hong Kong's Centre for Health Protection provides information about H1N1 in Hong Kong

## Introduction

Influenza A infection causes substantial morbidity and mortality each year [1]. Periodically, novel human strains emerge, spread rapidly, and cause increased incidence of infection, as was the case with the novel 2009 strain of H1N1 pandemic influenza (H1N1pdm) [2]. However, because many influenza infections are either asymptomatic or cause only mild symptoms, it is difficult to measure infection, rather than clinical disease, across a population [3]. With only clinical data, establishing robust rates of severe disease per infection is difficult. Also, it is not possible to establish traditional risk factors for infection. Hence, it is challenging to generate evidence-based advice for individuals and policy makers about the value of interventions designed to reduce the chance of infection, such as vaccination, social distancing, and other nonpharmaceutical interventions.

Previous community-based serological surveys of populations outside Hong Kong have established a broad consistent pattern for the 2009 influenza pandemic, namely, high rates of infection in school-aged children relative to younger adults and lower rates in older adults: Australia [4]–[7]; Belgium [8]; China [9]; Costa Rica [8]; England and Wales [10]; Germany [8]; India [11]; Japan [8]; New Zealand [12]; Scotland [13]; Singapore [14]; Thailand [15]; and the United States of America [8],[16]. Also, our own previous work has established a similar age-specific pattern of infection during the first wave in Hong Kong and per-infection mortality rates that escalated sharply with age, [17]. However, previous studies rely almost exclusively on noncohort designs and convenient recruitment, thus leaving a number of important issues unaddressed. For example, accurately estimating low attack rates in older individuals is challenging without paired sera, thus preventing robust estimates of severe disease in older individuals. Also, the absence of individual-level data other than age and sex prevents the investigation of straightforward hypotheses about possible risk factors for infection.

Here, we describe a longitudinal community cohort study of the main wave of the 2009 (H1N1) influenza pandemic in Hong Kong, with a design somewhat similar to the seroepidemiological components of the Tecumseh [18] and Seattle [19] studies. A substantive difference between our design and these two previous studies was that we attempted to recruit a representative sample of all households from a large well-mixed population, rather than restricting ourselves to a convenient sample of households with school-aged children.

## Methods

### Ethics Statement

All study protocols were approved by The Institutional Review Board of the University of Hong Kong/Hospital Authority Hong Kong West Cluster.

### Baseline Telephone Recruitment

Households were approached to take part in the study on the basis of their fixed-line telephone number. Numbers were obtained from two sources, either directly from random calling of residential landline numbers for Hong Kong (the direct group), or from a subgroup of participants that had already completed a parallel study of risk behaviors [20] and had indicated that they would be willing to be called again (the parallel group; Table S1). Members of the parallel study had themselves been recruited using random fixed-line phone numbers by the same team of call centre operatives.

**Figure 1:**
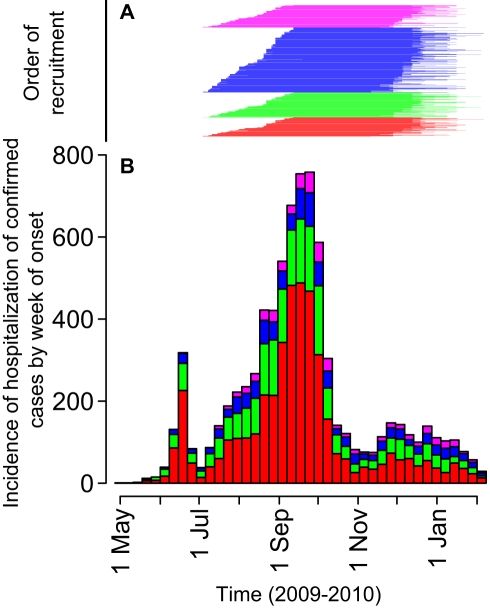
Timing of study recruitment relative to the time series of hospitalized cases in Hong Kong, by week of onset. Colors are coded for age groups in both charts: red, 3–19 y; green, 20–39 y; blue, 40–59 y; and magenta 60 y and older. (A) Shows the timing of recruitment of members of the study. (B) Shows the time series of hospitalized cases in Hong Kong, by week of onset.

We attempted to “bracket” the main wave of the pandemic by obtaining blood samples as soon as possible and then collecting follow-up samples when the peak of transmission had passed (Figure 1). The first baseline sample was taken on 4 July 2009 and the last on 19 September 2009. We started to follow-up individuals when clinical surveillance suggested that a substantial peak in transmission had passed. The first follow-up sample was taken on 11 November 2009 and the last taken on 6 February 2010. We invited participants to follow-up appointments in the order that they had attended for the baseline visit. However, if participants were unavailable for the initial appointment, we offered them as many further opportunities as required for them to attend (within the period of the study). The detailed pattern of recruitment timing, by individual, is presented in Figure S1.

When the phone was answered, we attempted to speak to an individual from the household who was at least 18 y old and who normally slept in the household for at least 5 nights per week. We explained the objectives of the study and asked the respondent if they and members their household were interested in participating. If the respondent agreed, we asked them to estimate the number of members of the household who would participate and we made an appointment for the household to visit the study clinic. The respondent was informed that at least one member of the household would be required to give a blood sample in order for the household to be eligible to enter the study.

### Clinic Visits

On arrival at the study clinic, each individual was given an information sheet and the opportunity to question a member of the study team. Informed consent was obtained individually for either full participation in the study, or for participation without giving a blood sample. For children aged 8–17, we obtained written consent from both the child and their parent or guardian. For children aged 2–7, written consent was obtained from the parent or guardian. A questionnaire was administered and ∼8 ml whole blood was obtained. Participating households were given a tympanic thermometer. Households were allowed to keep the thermometer at the end of the study. Participants who gave a blood sample were compensated with 100 HKD (≈US$13). Incentives were given as either supermarket vouchers (adults) or book tokens (children).

### Reporting of Symptoms

Participants were asked to report when any member of the household was experiencing two or more of: fever (>37.5°C, temperature measured only when a fever was suspected), cough, sputum, sore throat, runny nose, or myalgia. Participants were offered three methods of reporting. First, we asked them to phone the study team directly to report symptoms as soon as possible. Second, we asked them to fill out a paper diary with the day and type of the symptoms. Third, during a follow-up interview, we asked them if they had experienced any symptoms between baseline and follow-up and, if so, what symptoms they had experienced. For each mode of reporting, we constructed three types of symptomatic episode: acute respiratory infection (ARI, all reported episodes of symptoms), influenza-like illness (ILI, fever plus cough or sore throat), and fever alone.

### Laboratory Techniques

Blood samples were refrigerated at 4°C in the clinic and transferred (<1 h) using a cool box to the study laboratory later that evening. The next morning, samples were centrifuged at 1,500 rpm for 10 min and the sera extracted. The sera were frozen to −30°C for storage. For testing, sera were thawed and then heat inactivated at 56°C for 30 min. Replicate serum dilutions were mixed with 100 tissue culture infectious dose 50 (TCID50) of A/California/4/2009 (H1N1pdm) for 2 h and then transferred onto preformed monolayers of Madin-Darby Canine Kidney (MDCK) cells grown in 96-well microtitre plates. The plates were incubated at 37°C in 5% CO_2_ for 3 d. Neutralization of virus cytopathogenic effect (CPE) was observed under an inverted microscope to determine the highest serum dilution that neutralized ≥50% of the wells. A virus back titration, positive controls, and negative controls were included in each assay. The sensitivity of the test method was benchmarked using a standard positive control serum 09/194 provided by the National Institute for Biological Standards and Control, Centre for Health Protection, London. Neutralization tests, rather than hemagglutination tests, were chosen for assaying antibody responses to pandemic influenza H1N1 virus because neutralization tests are more sensitive for patients with virologically confirmed pandemic H1N1 infection [21].

Individuals were classified as seroconverters if there was a 4-fold or greater rise in their neutralization titre between baseline and follow-up. Initially, all samples were screened at dilutions of 1∶20 and 1∶40 with baseline and follow-up serum samples tested in parallel in the same set of assays. If exposure status or seroconversion status from the screening dilutions was unambiguous after these screening assays, no further titrations were performed. For all other pairs of sera, antibody titration was performed in 2-fold dilutions from 1∶10 to 1∶1,280.

### Data on Severe Cases

From the start of May 2009, patients admitted to public hospitals in Hong Kong with acute respiratory illness were routinely tested for H1N1pdm using reverse transcription (RT)-PCR. Every case for which a test was conducted was entered into an information management system administered by the Hospital Authority (eFlu). This system was integrated with the Hong-Kong–wide network for electronic notes and assigned a unique identifier based on Hong Kong identification numbers. In Hong Kong, 90% of inpatient bed days are in the public system [22]. We cross-referenced every positive test result with admissions and discharge data for the entire public system to identify individuals who had tested positive and subsequently been admitted to intensive care units (ICU) or who had died while in hospital. We removed duplicate hospital episode records, keeping the record closest to, but after, the positive test.

### Inferring Rates of Severe Disease

Estimating the number of infections per severe case would be straightforward if recruitment and follow-up had occurred during short periods of time and antibody titres rose immediately after infection. However, because of the rolling nature of recruitment and follow-up and the delay in the rise of antibody titres after infection, we developed a simple likelihood-based framework to estimate the number of infections per severe case for each age group, where a severe case could be an individual admitted to hospital, one admitted to an ICU, or a fatal case. Inference for a specific combination of age group and level of severity was independent of other combinations. Effectively, the proportion of an age group infected was equal to the number of severe cases, divided by the probability that an infection resulted in a severe case, expressed as a proportion of the total number of people in that age group (with adjustment for rising titres). Confidence intervals based on this approach reflect uncertainty arising from the size of the study and do not reflect other sources of uncertainty such as the variability in the speed with which antibody titres rise and the overall percentage of individuals whose antibodies rise after infection (we assumed 100%). Therefore, our results may slightly underestimate the number of infections. Details are given in Text S1.

### Simulation Study

In order to investigate alternate study designs and to validate our estimates of the rate of severe disease per infection, we developed a simulation of exactly the stochastic process assumed by the likelihood calculation above. For any given study protocol, we summed the number of actual severe cases between baseline and follow-up (for each individual, adjusting for rising titres) and then chose randomly between individuals being infected or not infected on the basis of the probability of severe infection. Thus, we could use the same likelihood framework to analyse results from the simulation studies as was used for the actual data.

## Results

### Study Population

Paired sera were obtained from 770 individuals living in 469 study households. Our response rates were (for households): 1.8% of all residential landlines selected (*n = *26,205) and 3.7% of all households in which an eligible adult completed the initial call (*n = *12,834) (Figure S2; Table S1). We compared the overall population of Hong Kong with the study group from which paired sera were obtained (Table S2). It is reassuring that our study population was representative of children aged 3–19 and adults of 60 y and older. The distribution of adults in the study between the ages of 20 and 59 was skewed towards older individuals, compared with the Hong Kong population in general. Women were more likely to take part in the study than were men, as were those with a bachelor's degree education or higher.

### Age and the Risk of Infection

The infection attack rate declined with increasing age. For those aged: 3–19, the attack rate was 39% (95% confidence interval 31%–49%); 20–39 y, 8.9% (5.3%–14.7%); 40–59 y, 5.3% (3.5%–8.0%); and 60 y or older, 0.77% (0.18%–4.2%). The attack rate in the oldest group had wide confidence intervals because only a single infection was observed in 131 participants. Differences in rates of seroconversion could not be explained by baseline titres, which were similar across age groups (Figure S3; Table S3). For example, five of 112 individuals ages 3–19 y had baseline titres of 1∶40 compared with eight of 131 individuals aged 60 y or older.

**Figure 2:**
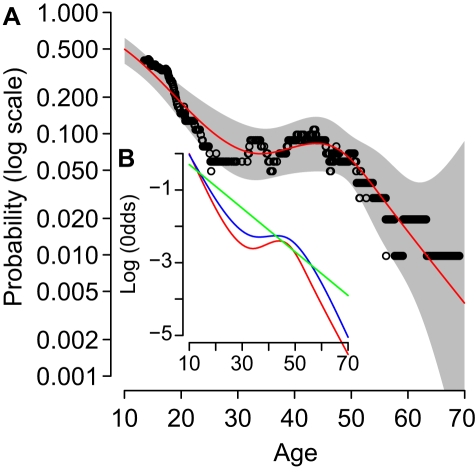
Age and risk of infection. (A) Shows the average probability of infection for median age (*x*-axis) in rolling windows of 100 study participants (black circles), the best-fit probability of infection (red line, univariate restricted cubic spline), and 95% confidence intervals (grey area). (B) Shows the log-odds, relative to age 1, for: the same best-fit univariate spline fit as in (A) (red); the spline age model adjusted for the presence of a child in the household (blue); and a linear model adjusted for the presence of a child (green, best-fitting model A in Table 1).

In order to fully capture the influence of age on the risk of infection, we used the Akaike Information Criterion (AIC) to compare three alternative regression models: 20-y age classes, AIC = 414.1; linear age, AIC = 413.0; and a restricted cubic spline model, AIC = 407.7. We considered spline fits with between 3 and 8 knots: the 5-knot curve was best able to explain the data. The fitted spline function corresponded well with age-based rolling average of infection incidence and shows: a sharp drop in risk of infection ages older than school age, followed by a plateau for middle ages, before another sharp drop for older adults (Figure 2A).

The presence of a child in the household explained the plateau in the age-risk of infection in these data. We adjusted the spline model using a binary variable for the presence or absence of a child in the household and compared the shape of the odds ratio curve (Figure 2B, blue line, AIC = 406.1) with the unadjusted model: the odds ratio curve was more linear, with less pronounced turning points. Therefore, we also fitted the linear age model, adjusted for the presence or absence of a child. We found that the adjusted linear model was a more parsimonious explanation for the data (Figure 2B, green line; Table 1, model A, AIC = 405.5, ΔAIC = 0). The age-adjusted odds ratio for infection for those in a household without children was 0.39 (0.21–0.73), relative to those living in households with children.

**Table 1:** Risk factors for infection with 2009 H1N1 pandemic influenza for 667 participants of the study for whom paired sera were tested and for whom complete information was available.

Risk Factor	ΔAIC^a^	Value	Univariate Models		Model A		Model B		Model C	
			OR	95% CI	OR	95% CI	OR	95% CI	OR	95% CI
Age	7.5	1 y old	1.0	—	1.0	—	1.0	—	1.0	—
		Per year older than 1	0.93	0.92–0.95	0.94	0.93–0.96	0.94	0.93–0.96	0.94	0.93–0.96
Child in house	59.6	Present	1.0	—	1.0	—	1.0	—	1.0	—
		Not present	0.18	0.098–0.32	0.39	0.21–0.75	0.4	0.21–0.75	0.4	0.21–0.75
Sex	101.9	Female	1.0	—	—	—	1.0	—	1.0	—
		Male	1.3	0.79–2.0	—	—	0.95	0.56–1.6	0.94	0.55–1.6
District	96.4	HK Island	1.0	—	—	—	1.0	—	1.0	—
		KLN East	1.2	0.42–3.2	—	—	1.2	0.39–3.5	1.2	0.40–3.6
		KLN West	1.9	0.73–4.9	—	—	2.7	0.96–7.8	2.8	0.98–7.9
		NT East	3.1	1.4–6.9	—	—	2.6	1.1–6.2	2.5	1.1–6.1
		NT West	1.9	0.81–4.7	—	—	1.5	0.56–3.8	1.4	0.55–3.7
Vaccination 2008/2009	102.5	Not vaccinated	1.0	—	—	—	1.0	—	1.0	—
		Vaccinated	1.2	0.66–2.1	—	—	1.5	0.79–3.0	1.4	0.55–3.7
Recruitment	99.8	Direct	1.0	—	—	—	—	—	1.0	—
		Parallel	1.5	0.95–2.4	—	—	—	—	1.3	0.77–2.2
**ΔAIC** b					**0**		**2.5**		**3.5**	

Data for one individual missing vaccination status and for another two were missing education status.

a AIC for individual univariate models relative to that of the best fit multivariate model A.

b AIC for mutually adjusted multivariate models, relative to that of Model A.

CI, confidence interval; HK, Hong Kong; KLN, Kowloon; NT, New Territories (outlying islands included in NT West); OR, odds ratio.

### Other Risk Factors

We had substantive interest in six other potential risk factors, in addition to age and the presence or absence of a child in the household. In order to efficiently prioritize model selection, we calculated the AIC for all possible regression models (63 nonempty subsets of six risk factors) combined with linear age and the presence or absence of a child in the household. None of the 63 models had a lower AIC than model A. Only three additional risk factors appeared in models not substantially different from model A on the AIC scale (ΔAIC <3): district of residence, sex, and status for 2008/2009 influenza vaccination (Table 1). Even though univariate analyses suggested association in some cases, the following three risk factors did not appear in models close to the best model: household size (best ΔAIC = 7.4), profession (best ΔAIC = 6.4), and level of education (best ΔAIC = 5.4).

Residents of New Territories East had an increased risk of infection during the study period, even after adjusting for other risk factors of interest (Table 1, model B, ΔAIC = 2.5), with an odds ratio of 2.6 (1.1–6.2) relative to residents of Hong Kong Island. Although sex was included in a number of well-fitting models, the odds ratio for males relative to females was close to unity in the adjusted model, suggesting that correlation between sex and other risk factors in the study group was somewhat different from that in the wider population. Although not statistically significant, estimates of the adjusted odds ratio for vaccine status suggest that those who reported being vaccinated in 2008/2009 were at an increased risk of infection.

In order to control for possible bias from the two alternate sources of recruitment (the direct group or the parallel group), we included the source of recruitment as a possible confounder in a final mutually adjusted regression model, model C. This model scored slightly worse on the AIC scale than did model B (ΔAIC = 3.5), with only very minor changes in estimated odds ratios and confidence intervals. Although recruitment from the parallel group was associated with an increased risk of infection, the estimated odds ratio was not significantly different from unity.

We also considered a baseline titre of 1∶40 or greater as a risk factor for infection (despite this variable being a component of the outcome variable, Table S3). Even though a low baseline titre was protective, the small number of raised titres observed ensured that this variable had little explanatory power (ΔAIC for model A with baseline titre added as a binary variable  = 1.1).

Individual-level data from the serological survey are provided as Dataset S1 with the fields defined in Table S4.

### Symptoms

**Figure 3:**
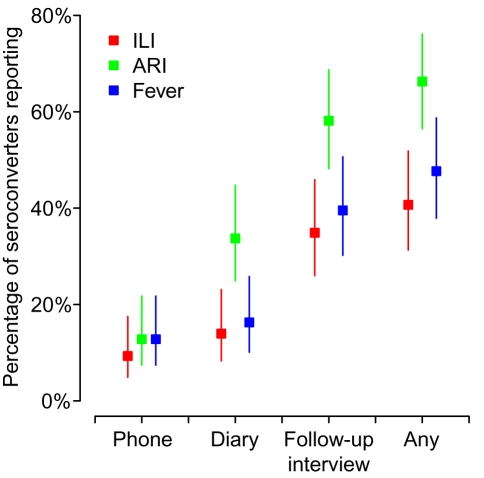
Absolute levels of symptom reporting. Three different definitions of symptoms were used: ILI, acute respiratory infection, or fever (see main text for details). Symptoms were reported by one of: study participants phoning into the study phone line, by symptom diary, or at follow-up interview. We also report an all-inclusive rate: the percentage of seroconverters that reported symptoms by any of the three modes. 95% confidence bounds are based on the binomial distribution.

Rates of reported symptoms were low, but varied substantially by definition and by mode of reporting (Figure 3). In general, when reporting by phone or by symptom diary, between 10% and 20% of seroconverters reported having experienced symptoms. The only exception was the reporting of ARI by diary, which was considerably higher. Rates of reporting by follow-up interview were considerably higher. Febrile symptoms for seroconverting children were reported by phone more often than for adults who seroconverted (Table 2). Of the 41 children seroconverters in the study, ten reported by phone that they had experienced a fever and, of those, seven reported ILI. In contrast only one of 40 adult seroconverters in the study had telephoned us to report a febrile illness (and also ILI).

**Table 2:** Symptoms reported by study participants by infection status, symptom definition, and method of reporting.

Reporting Method	Seroconverted			Nonseroconverted		
	Age Groups		*p*-Value	Age Groups		*p*-Value
	*n* 0–18 y (%)	*n* 19+ y (%)		*n* 0–18 y (%)	*n* 19+ y (%)	
***n*** ** Laboratory tested (%)**	41 (48%)	45 (52%)		57 (8%)	627 (92%)	
**ILI** a						
Phone	7 (17%)	1 (2%)	0.059^b^	2 (4%)	12 (2%)	0.335^b^
Diary	9 (22%)	3 (7%)	0.122^b^	3 (5%)	10 (2%)	0.093^b^
Follow-up interview	19 (46%)	11 (24%)	0.206^c^	7 (12%)	44 (7%)	0.289^c^
Any of the above source	24 (59%)	11 (24%)	0.059^c^	11 (19%)	57 (9%)	0.054^c^
**Acute respiratory infection** d						
Phone	9 (22%)	2 (4%)	0.052^b^	4 (7%)	27 (4%)	0.327^b^
Diary	18 (44%)	11 (24%)	0.260^c^	9 (16%)	80 (13%)	0.716^c^
Follow-up interview	24 (59%)	26 (58%)	0.888^c^	26 (46%)	192 (31%)	0.143^c^
Any of the above source	31 (76%)	26 (58%)	0.539^c^	29 (51%)	204 (33%)	0.084^c^
**Fever** e						
Phone	10 (24%)	1 (2%)	0.009^b^	2 (4%)	14 (2%)	0.637^b^
Diary	9 (22%)	5 (11%)	0.387^c^	5 (9%)	20 (3%)	0.059^b^
Follow-up interview	21 (51%)	13 (29%)	0.234^c^	8 (14%)	53 (9%)	0.302^c^
Any of the above source	27 (66%)	14 (31%)	0.084^c^	13 (23%)	67 (11%)	0.034^c^

a ILI is defined as fever (temperature of 37.5°C or above) + cough or sore throat.

b Fisher's exact test.

c Chi-squared test.

d Defined as any two of fever^e^, cough, phlegm, sore throat, running nose, and myalgia.

e Temperature of 37.5°C or above.

### Rates of Severe Disease

**Figure 4:**
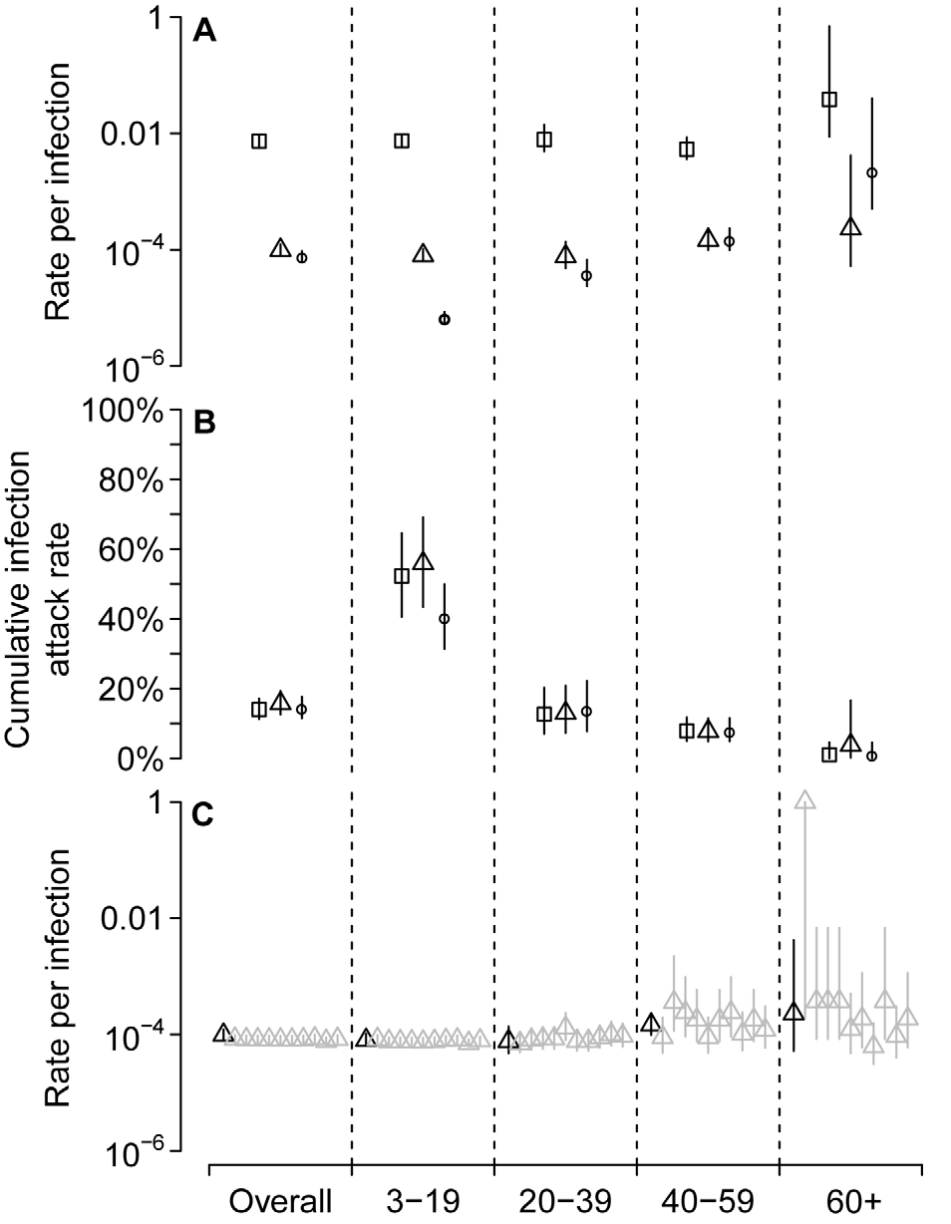
(A) Overall and age-specific estimated rates of severe disease per infection; squares for hospitalization, triangles for admission to ICUs, and circles for death. (B) Estimated cumulative attack rates for infection up to the end of January 2010. Three separate estimates of cumulative infection attack rate are given for each age group, on the basis of the three levels of severity, with symbols as per (A). (C) Comparison of estimates of rates of ICU admission per infection from the current study (black triangles, as per (A)) with estimates of the same statistic from ten simulations of an alternate, nonbracketing, study design (see text).

The overall rate of confirmed H1N1pdm-associated deaths was 7.6 (6.2–9.5) per 100,000 infections. Rates of severe disease increased with age (Figure 4A). Although rates of hospitalization per infection were not substantially different for the younger three age groups (∼1%), individuals aged 60 and older were at slightly increased risk. However, rates of mortality increased substantially with age. The risk of death per infection for 3–19 y olds was 1.3 (1.0–1.7) per 100,000 while the risk for individuals aged 60 or older was 220 (50–4,000) per 100,000. Wide confidence intervals for 60 y and older were driven by the single observed infection in that group. However, the general trend of rapidly increasing mortality with age can be seen clearly across the other adult age groups.

### Cumulative Attack Rate up to End January 2010

Although we aimed to obtain bracketing sera (Figure 1), there was substantial transmission outside of the period of our study. Therefore, we used the total number of confirmed severe cases to estimate the age-specific cumulative infection attack rates for a longer period of time than was captured by our study (Figure 4B). Between 27 April 2009 and 7 February 2010, in Hong Kong, there were 7,981 hospitalizations, 109 admissions to ICU, and 70 deaths among laboratory confirmed cases of H1N1pdm. Using the rates of severe disease per infection, these totals suggest cumulative infection attack rates of: 14% (11%–17%), based on hospitalization; 16% (13%–19%), based on ICU admission; and 14% (12%–18%), based on deaths.

### Simulation of a Nonbracketing Design

We simulated an alternate trial design in which 500 samples were obtained from each of the four age groups during the week containing 1 June 2009 and the 500 follow-up samples were obtained from each age group during the week containing 30 September 2009 (Figure 4C). In this trial design, fewer infections and many fewer severe cases were observed. However, despite the reduced power of the alternate design, overall rates of severity are well estimated and the pattern of increasing severity with age is readily apparent. Even though no infections were observed in some of the simulated studies in the 60 and older age class, it was still possible to establish a relatively high lower bound for rates of severe disease. Although we present results for ICU as an example, the results for admission to hospital and death are not substantively different.

## Discussion

The main wave of the 2009 (H1N1) pandemic infected many more children than it did adults. These differences are not explained by baseline antibody titres to H1N1pdm, but could be explained partly by social mixing patterns of the population in these different age strata. However, given that social mixing patterns within the 20–60-y age range do not exhibit substantial variation [23], and that we have controlled for the presence of a child in the household, it is plausible that increasing age leads to decreased susceptibility independently of mixing and titres to H1N1pdm, possibly as a result of repeated seasonal influenza infections, but by a mechanism not detectable by assays for neutralizing antibody. Whether this reflects antibody that protects by mechanisms other than neutralization, such as antibody-dependent cell cytotoxicity or cell-mediated immunity, remains worthy of investigation.

While older adults had low infection rates, those individuals infected developed severe disease much more frequently. Further, our results suggest that individuals over 60 y experience very high absolute rates of severe outcomes, with approximately one reported and positively tested death for every 200 infections. If continuing waves of H1N1pdm infection are driven by antigenic drift, and if that drift decreases the efficiency of the cross-protection currently possessed by older adults, it is likely that future waves could have higher overall mortality than initial waves. Surveillance of clusters of severe disease in older adults should be prioritized because this may be the first clear signal of a significant antigenic evolutionary event. The efficacy of alternate vaccine formulations in preventing infection in older individuals should be assessed as a matter of priority [24].

The low rate of ILI reported by phone and symptom diary for seroconverters in this study is consistent with results from an independent parallel study of household contacts of children in Hong Kong [21]. However, much higher rates of symptoms were reported in military personal in Singapore [25]. These differences may be driven by the age distributions of the different cohorts. The Singapore cohort was much younger: our comparison of symptom reporting by age suggests that reduced level of symptom reporting observed in adults was likely due to a reduced rate of experiencing symptoms, rather than just to a reduced propensity to report. The proportion of uninfected children that reported febrile illness was similar to that of uninfected adults. However, our results do suggest that adults are slightly less likely to report symptoms during a follow-up interview than are children. In general, rates of reporting were much higher but less specific when participants were asked a direct question during the follow-up interview compared with more passive symptom diaries or participant call-in. Future studies attempting to address rates of illness associated with influenza infection should attempt more intensive prospective follow-up of participants to minimize potential recall bias.

Our study has a number of limitations. Firstly, we did not measure incidence in children aged 2 and lower, who are much more likely to be admitted to hospital for acute respiratory infection than other age groups, but less likely to be infected with pandemic influenza than older children [26]. Given our focus on the use of paired sera, this shortcoming was unavoidable. The incorporation of data from cross-sectional samples from young children is the topic of ongoing investigation. Also, our cases are defined by an observed 4-fold rise in titre, which may not have occurred for all infections; and we will not have captured all severe cases as some H1N1pdm cases will have entered the private hospital system (although this is likely to have occurred much more rarely than the average rate of ∼10% for all types of admission). We suggest that the combined effect of imperfect test sensitivity and imperfect severe case matching will have generated only minor biases in our estimates of infection rates and severe disease rates and that the two effects will have acted in opposite directions.

In order to extrapolate from the period of our study to the full period of the pandemic in Hong Kong, we made the assumption that the testing process for individuals who became hospitalized was consistent. This is almost certainly not the case for all hospitalized individuals. In particular, anecdotal evidence suggests that less severe hospitalized cases were less likely to be tested for H1N1pdm after the end of September. Analysis of the rate of admission to ICU per positive hospital admission supports the anecdotal evidence (unpublished data). Therefore, it is reassuring that estimates of the overall and age-specific attack rates based on the three different outcome measures (hospitalization, admission to ICU, and death) are largely consistent.

We cannot exclude the possibility of substantial sampling bias in our serological survey. We were only able to successfully obtain paired sera from an average of 1.6 individuals in ∼2% of households initially identified by random telephone number selection. Although similar in many respects, after using common demographic characteristics to compare the study population with the wider Hong Kong population (age, sex, district, and education), we cannot exclude the possibility that individuals more likely to take part in our study had a different probability of infection than the population at large. However, we suggest that the potential impact of sampling bias in our results (and the value of evidence presented here in general) should be assessed on a result-by-result basis against the background of other reported community surveys of the 2009 influenza pandemic.

For the 2009 Hong Kong pandemic season, the current results add substantially to our earlier work [17] in a number of ways. Using an entirely different sampling scheme, the current study confirms the general pattern of sharply decreasing age-specific rates of infection for a similar period of the epidemic, for the age ranges contained in both studies: thus strengthening the case for rapid cross-sectional serological studies on the basis of convenient samples [17] at the same time as suggesting that serious sampling biases were not present in either study. Also, to a certain extent, multiple recruitment groups in the current study provided a proxy for propensity to take part: those recruited via the parallel study had already agreed to complete one telephone questionnaire and to be contacted again for other studies. Having agreed for a third time to take part, by enrolling in the main serological survey, it seems reasonable to assume that parallel study recruits are from a subset of the population more likely to take part in this type of study. Although our univariate estimate of the odds ratio for the parallel recruitment group was greater than one, compared with the direct group, the inclusion of recruitment source as a covariate did not improve the parsimony of our multivariate regression model. Also, the strength of the odds ratio for the parallel group in the univariate model was reduced substantially when adjusting for our epidemiological variables of interest. Therefore, although it is certainly possible that propensity to take part in the paired serological study was correlated with the risk of infection, comparison with our previously published cross-sectional study and comparison of our two recruitment groups suggests that sample bias was of considerably lesser influence on infection than variables we were able to measure directly, such as age and the presence of a child in the household.

The current study, by recruiting from a wide age range using the same sampling framework and a paired sera outcome, allows us to add to the available literature in a number of other ways. We present important data on infection rates and severity for those aged 60 y and older that were not reported in our previous study [17]: even the single observed 4-fold rise in titre out of 131 paired samples is valuable. In an age group typically at high risk from influenza morbidity and mortality, these data allow an informative upper bound for the absolute risk of infection and, hence, an informative lower bound for the absolute risk of severe outcomes. Without paired samples, obtaining accurate bounds for estimates of low rates of incidence from cross-sectional samples is problematic. When trying to estimate low rates of incidence with cross-sectional data, statistical noise becomes significant in the numerator: to overcome this noise, large sample sizes are required. Good evidence for high absolute risk in a particular age group may be of substantial public health value for the prioritization of interventions.

With good data on other potential risk factors for infection, we were able to show how the presence of a child in the household could explain an apparent age plateau in risk of infection, while variables such as education and profession did not appear to be risk factors once adjusted for age. This type of traditional risk-factor analysis is not possible with unlinked samples for which only the following variables are usually available: age, sex, and clinic location. Similarly, our analysis of home district (using only a single clinic location) suggests that micro-scale spatial heterogeneities persisted for longer than might have been expected in a large well-connected population. For the period of our study, residents of one district (New Territories East) appeared to be at substantially greater risk of infection than were residents of other districts. It is possible that the overall level of transmission was higher in that one district than in other districts, or that the epidemic occurred sooner there than it did elsewhere. Further, it seems possible that, in Hong Kong, spatial decorrelation took a long time to occur or never did occur. Individual-based models of respiratory infections, parameterized with the commuting patterns of adults [27], and also those parameterized with explicit school locations [28],[29], suggest much more rapid spread at small scales in large populations. Had the pandemic strain been more severe, good knowledge of small-scale spatiotemporal patterns could have been of value in optimizing the provision of key health care facilities and the timing of rolling school closures.

Our results can be compared with serology-based studies of influenza incidence in other populations during the 2009 (H1N1) pandemic [4]–[16]. In England and Wales, a study of cross-sectional clinical samples found substantial increases in the proportion of younger children with titres 1∶32 or greater between a 2008 baseline (*n = *1,403) and sample taken in September 2009 (*n = *1,954), thus giving valuable early evidence that the infection attack rate was high in some age groups and, hence, that the rate of severe cases per infection in the most affected age groups was likely to be low [10]. As already mentioned above, the consistency of our results for Hong Kong between the current study and our previous cross-sectional study [17] validates the use of convenient clinical samples during the early stages of a pandemic as a useful tool for the estimation of incidence in high-incidence groups. However, a high degree of cross-reactivity in hemagglutination inhibition assays in sera from adults in many studies introduced considerable statistical noise and prevented reliable estimates of attack rates in older age groups. In Singapore, sera were collected from four groups: an existing sample of healthy adults (*n = *838), military personnel (*n = *1,213), staff from an acute care hospital (*n = *558), and staff and residents of a long-term care facility (*n = *300) [14]. Although an overall infection attack rate of 13% was observed in this study, it is difficult to generalize these results because no subgroup contained school-aged children and many of the infection events occurred in the military substudy. 

It is more difficult to compare our community-wide results with historical studies such as the Tecumseh [18] and Seattle [19] study. Both were designed to efficiently obtain viral samples from households with children and, therefore, did not attempt to recruit from childless households. Also, information on their precise sampling framework for households with children is difficult to obtain. However, future analyses of household-level data from the current study and follow-up waves should permit a like-for-like comparison between the subgroups in the Hong Kong study and canonical historical studies of respiratory infection.

Our simulation results show that a larger paired-sera cohort study with a shorter follow-up period could have generated—more rapidly—similar data to those presented here. We suggest that this revised design would be a valuable addition to revised pandemic preparedness plans for a small subset of large well-connected global cities. Sentinel hospitals could be established in early-affected populations to help ensure that the testing process remains consistent for ICU cases throughout the epidemic curve. Given that (a) many believe the 2009 response to have been overzealous and (b) the severity of the next pandemic strain is not known, there appears to be a substantial risk that the public health impact of the next pandemic will be underestimated. Therefore, revised preparedness plans should prioritize reactive studies that can rapidly and reliably distinguish between 2009 (H1N1)-like strains (∼1∶10,000 infection fatality rate) and more severe pandemics. If the next pandemic strain were similar in all other respects, but had an infection fatality rate of ∼1∶1,000; we could reasonably expect peak demand on key health care services such as ICU to be ten times greater than that observed during 2009/2010 [30].

## Supporting Information

Dataset S1 Individual-level data from the serological study (see Table S4 for definition of fields).
(0.05 MB CSV)

Figure S1. The timing of recruitment and follow-up for all 770 individuals for which baseline and follow-up samples were available. A small amount of random noise was added to both the *x* and *y* coordinates so that individuals with the same baseline and follow-up dates can be distinguished.
(0.07 MB PDF)

Figure S2. Flow chart of study recruitment. See Table S1 for the number of individuals at each stage in the process.
(0.08 MB PDF)

Figure S3 Neutralization titres against H1N1pdm. The location of each pie chart indicates neutralization tire at baseline (*x*-axis) and at follow-up (*y*-axis). The radius of each pie chart is proportional to the number of individuals with a particular combination of baseline and follow-up titres, on a log scale (base 10, see legend). The color mix within each size pie chart indicates the mix of age groups with a specific combination of baseline and follow-up titres. Seroconverters are above and to the left of the diagonal grey line. Colors are coded for age groups: red, 3-19 y; green, 20–39 y; blue, 40–59 y; and magenta 60 y and older.
(0.18 MB PDF)

Table S1 Recruitment of households by random dialing and from parallel attitude's study.
(0.06 MB PDF)

Table S2. Characteristics of the study population compared with the population of Hong Kong.
(0.08 MB PDF)

Table S3 The relationship between age group, baseline neutralization titre, and seroconversion status.
(0.06 MB PDF)

Table S4 Dictionary of field names for Dataset S1 (data from serological study).
(0.06 MB PDF)

Text S1 A statistical model for severity
(0.18 MB PDF)

## Abbreviations

AIC: Akaike Information Criterion
H1N1pdm: H1N1 pandemic influenza
ICU: intensive care unit
ILI: influenza-like illness
