# Supplementary material for: Epidemiological Characteristics of 2009 (H1N1) Pandemic Influenza Based on Paired Sera from a Longitudinal Community Cohort Study
Source: PLoS Med. 2011 Jun 21;8(6):e1000442. doi: 10.1371/journal.pmed.1000442 (PMC3119689; doi:10.1371/journal.pmed.1000442)
Supplement: Figure S1. — The timing of recruitment and follow-up for all 770 individuals for which baseline and follow-up samples were available. A small amount of random noise was added to both the x and y coordinates so that individuals with the same baseline and follow-up dates can be distinguished. (0.07 MB PDF) [file pmed.1000442.s002.pdf]

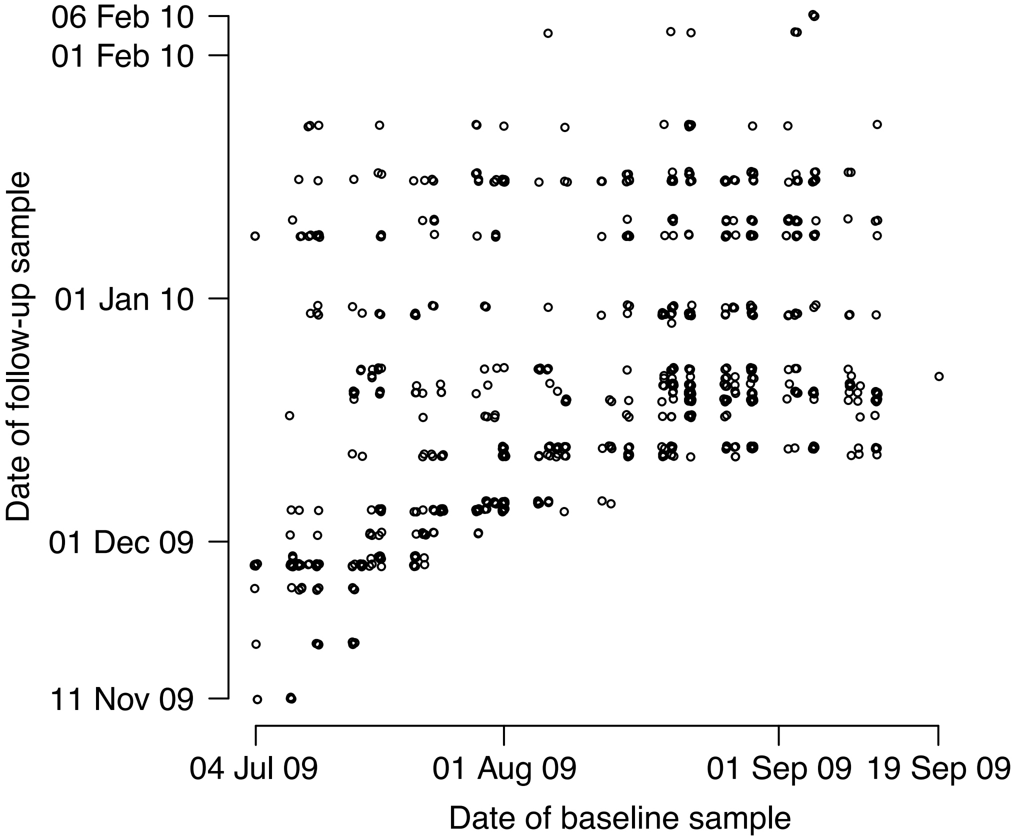

Figure S1. The timing of recruitment and followup for all 770 individuals for which baseline and follow-up samples were available. A small amount of random noise was added to both the x and y coordinates so that individuals with the same baseline and follow-up dates can be distinguished.
