# Supplementary material for: Epidemiological Characteristics of 2009 (H1N1) Pandemic Influenza Based on Paired Sera from a Longitudinal Community Cohort Study
Source: PLoS Med. 2011 Jun 21;8(6):e1000442. doi: 10.1371/journal.pmed.1000442 (PMC3119689; doi:10.1371/journal.pmed.1000442)
Supplement: Figure S2. — Flow chart of study recruitment. See Table S1 for the number of individuals at each stage in the process. (0.08 MB PDF) [file pmed.1000442.s003.pdf]

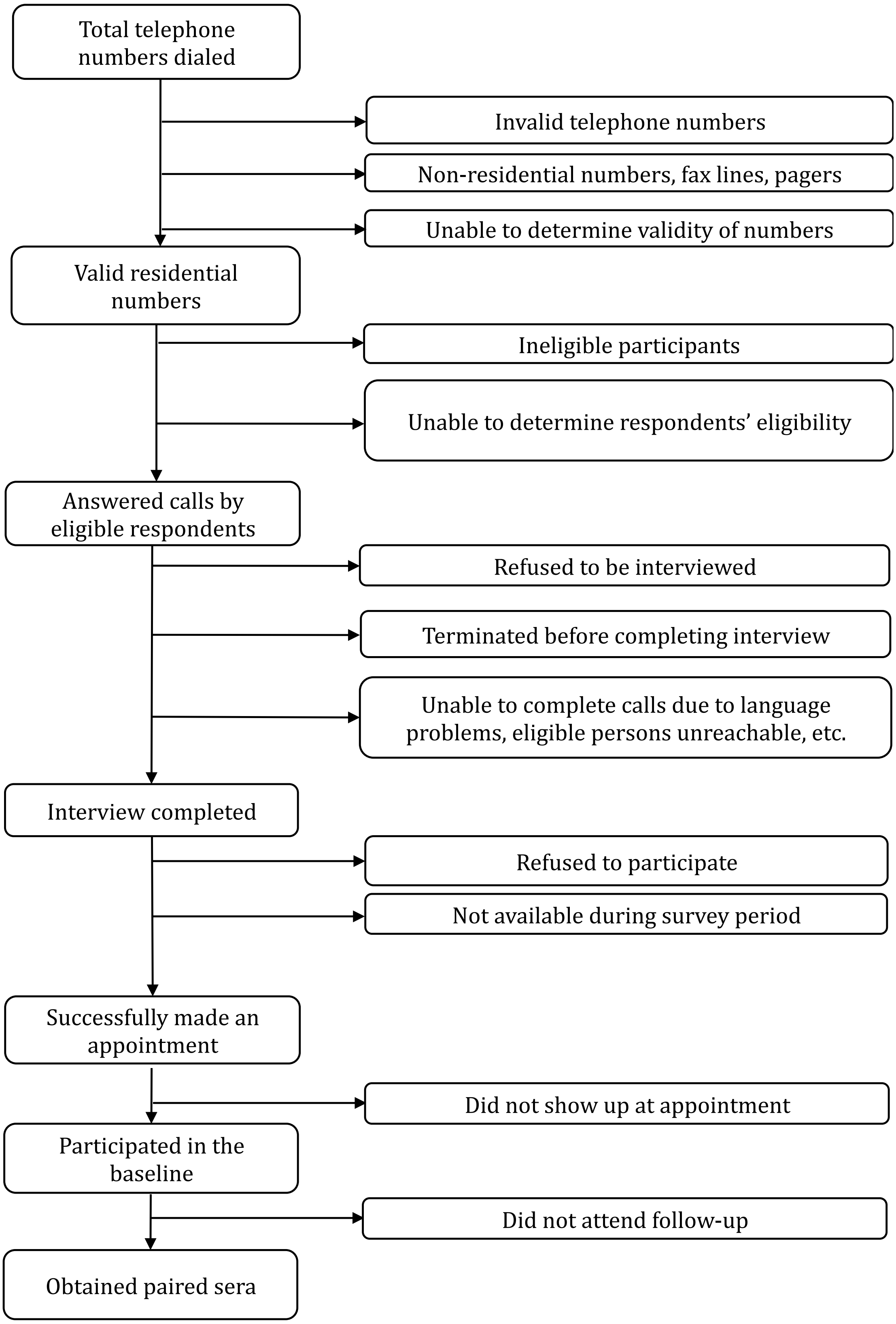

Figure S2. Flow chart of study recruitment. See Supporting Table 1 for the number of individuals at each stage in the process.
