# Supplementary material for: Epidemiological Characteristics of 2009 (H1N1) Pandemic Influenza Based on Paired Sera from a Longitudinal Community Cohort Study
Source: PLoS Med. 2011 Jun 21;8(6):e1000442. doi: 10.1371/journal.pmed.1000442 (PMC3119689; doi:10.1371/journal.pmed.1000442)
Supplement: Figure S3 — Neutralization titres against H1N1pdm. The location of each pie chart indicates neutralization tire at baseline (x-axis) and at follow-up (y-axis). The radius of each pie chart is proportional to the number of individuals with a particular combination of baseline and follow-up titres, on a log scale (base 10, see legend). The color mix within each size pie chart indicates the mix of age groups with a specific combination of baseline and follow-up titres. Seroconverters are above and to the left of the diagonal grey line. Colors are coded for age groups: red, 3-19 y; green, 20–39 y; blue, 40–59 y; and magenta 60 y and older. (0.18 MB PDF) [file pmed.1000442.s004.pdf]

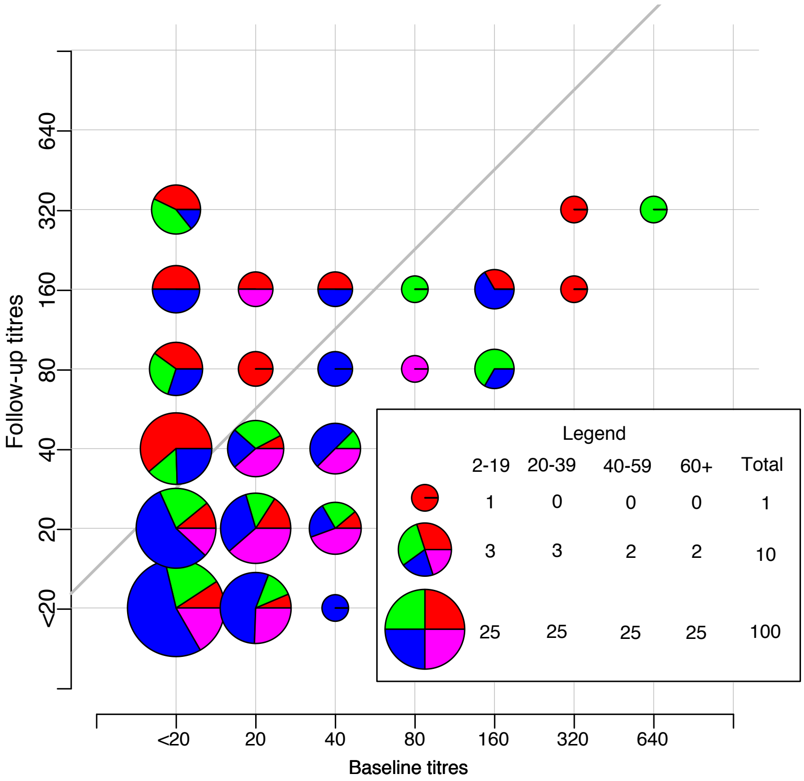

Figure S3. Neutralization titres against H1N1pdm. The location of each pie chart indicates neutralization tire at baseline (x-axis) and at follow-up (y-axis). The radius of each pie chart is proportional to the number of individuals with a particular combination of baseline and follow-up titres, on a log scale (base 10, see legend). The color mix within each size pie chart indicates the mix of age groups with a specific combination of baseline and follow-up titres. Seroconverters are above and to the left of the diagonal grey line. Colors are coded for age groups: red, 3-19 years; green, 20-39 years; blue, 40-59 years and magenta 60 years and older.
