# Supplementary material for: Epidemiological Characteristics of 2009 (H1N1) Pandemic Influenza Based on Paired Sera from a Longitudinal Community Cohort Study
Source: PLoS Med. 2011 Jun 21;8(6):e1000442. doi: 10.1371/journal.pmed.1000442 (PMC3119689; doi:10.1371/journal.pmed.1000442)
Supplement: Table S1 — Recruitment of households by random dialing and from parallel attitude's study. (0.06 MB PDF) [file pmed.1000442.s005.pdf]

**Table S1.** Recruitment of households by random dialing and from parallel attitude's study[1].

| **Status** | **Direct** | **Parallel** | **Total** |
|---|---|---|---|
| Total telephone numbers dialed | 41,974 | 2,183 | 44,157 |
| (-) Invalid telephone numbers | 13,221 | 25 | 13,246 |
| (-) Non-residential numbers, fax lines, pagers | 4,552 | 26 | 4,578 |
| (-) Unable to determine validity of numbers | 115 | 13 | 128 |
| (=) Valid residential numbers | 24,086 | 2,119 | 26,205 |
| (-) Ineligible participants | 47 | 648 | 695 |
| (-) Unable to determine respondent's eligibility | 11,772 | 679 | 12,451 |
| (=) Answered calls by eligible respondents | 12,267 | 792 | 13,059 |
| (-) Refused to be interviewed | 160 | 13 | 173 |
| (-) Interviews terminated before completion | 32 | 3 | 35 |
| (-) Unable to complete calls due to language | 9 | 8 | 17 |
| (=) Interview completed | 12,066 | 768 | 12,834 |
| (-) Refused to participate | 2,525 | 2 | 2,527 |
| (-) Not available during survey period | 9,007 | 421 | 9,428 |
| (=) Successfully scheduled an appointment | 534 | 345 | 879 |
| (-) Did not show up at appointment | 178 | 113 | 291 |
| (=) Participated in baseline | 356 | 232 | 588 |
| (-) Did not attend follow-up | 71 | 48 | 119 |
| (=) Participated in baseline and follow-up | 285 | 184 | 469 |

[1]See Figure S1 for flow diagram of stages of recruitment.
