# Supplementary material for: Epidemiological Characteristics of 2009 (H1N1) Pandemic Influenza Based on Paired Sera from a Longitudinal Community Cohort Study
Source: PLoS Med. 2011 Jun 21;8(6):e1000442. doi: 10.1371/journal.pmed.1000442 (PMC3119689; doi:10.1371/journal.pmed.1000442)
Supplement: Table S2. — Characteristics of the study population compared with the population of Hong Kong. (0.08 MB PDF) [file pmed.1000442.s006.pdf]

Table S2. Characteristics of the study population compared with the population of Hong Kong.

Characteristic	Value	Hong Kong (000s)[4]	Study (n=770, with 95% binomial confidence bounds)[4]
Age[1]	3 - 19	1,154 (16.8%)	112 (14.5% , [12.2% , 17.2%])
	20 - 39	2,105 (30.6%)	146 (19.0% , [16.4% , 21.9%])
	40 - 59	2,367 (34.4%)	381 (49.5% , [46.0% , 53.1%])
	60 and older	1,260 (18.3%)	131 (17.0% , [14.5% , 19.9%])
	Missing	0	0
Sex[1]	Male	3,300 (47.0%)	307 (39.9% , [36.5% , 43.4%])
	Female	3,726 (53.0%)	463 (60.1% , [56.7% , 63.6%])
	Missing	0	0
Education[2]	Kidergarten or lower	423 (7.1%)	16 (2.1% , [1.3% , 3.4%])
	Primary	1,084 (18.3%)	128 (16.7% , [14.2% , 19.4%])
	Secondary	3,505 (59.1%)	479 (62.4% , [58.8% , 65.6%])
	Bachelor or above	915 (15.4%)	145 (18.9% , [16.3% , 21.8%])
	Missing	711	2
Occupation[2,3]	Managers	567 (8.6%)	109 (14.2% , [11.9% , 16.8%])
	Clerks	1,110 (16.8%)	84 (10.9% , [8.9% , 13.3%])
	Service	837 (12.7%)	47 (6.1% , [4.6% , 8.0%])
	Elementary	848 (12.8%)	52 (6.8% , [5.2% , 8.8%])
	Students	1,369 (20.7%)	142 (18.4% , [15.9% , 21.4%])
	Home makers and others	1,881 (28.4%)	336 (43.6% , [40.2% , 47.2%])
	Missing	24	0
Family Size[2]	1	368 (5.5%)	32 (4.2% , [3.0% , 5.8%])
	2	1,071 (16.1%)	140 (18.2% , [15.6% , 21.1%])
	3	1,551 (23.4%)	207 (26.9% , [23.9% , 30.2%])
	4	2,020 (30.4%)	254 (33.0% , [17.4% , 23.0%])
	5	1,069 (16.1%)	105 (13.6% , [11.4% , 16.3%])
	6 or more	557 (8.4%)	32 (4.2% , [3.0% , 5.8%])
	Missing	0	0
District[2]	Hong Kong Island	1,268 (19.1%)	145 (18.8% , [16.3% , 21.8%])
	Kowloon East	1,011 (15.2%)	128 (16.6% , [13.9% , 19.2%])
	Kowloon West	643 (9.7%)	112 (14.5% , [12.3% , 17.2%])
	NT East	1,588 (23.9%)	228 (29.6% , [26.5% , 33.0%])
	NT West and marine	1,988 (30.0%)	157 (20.4% , [17.7% , 23.4%])
	Missing	137	0

[1]Age (n= 6,886 and sex (n=7,026), corrected to a thousand, are projected estimates from the Census and Statistics Department of the Hong Kong SAR Government, which is according to the Population By-census conducted, 2006.

[2]Education, occupation, family size and district are from the By-census conducted in 2006, and the total of the resident population was 6,636 thousand (totals do not always sum due to rounding).

[3]Some classes were aggregated: for "managers", managers, administrators and professionals; for "clerks", associate professionals and clerks; for "service workers", service workers, shop sales workers, craft and related workers; for "elementary", plant and machine operators and assemblers, elementary occupations, and skilled agricultural and fishery workers; "home-makers and others", home-makers, retirees, unclassified, and unemployed.

[4]Percentages do not include missing values.
