# Supplementary material for: Epidemiological Characteristics of 2009 (H1N1) Pandemic Influenza Based on Paired Sera from a Longitudinal Community Cohort Study
Source: PLoS Med. 2011 Jun 21;8(6):e1000442. doi: 10.1371/journal.pmed.1000442 (PMC3119689; doi:10.1371/journal.pmed.1000442)
Supplement: Table S3 — The relationship between age group, baseline neutralization titre, and seroconversion status. (0.06 MB PDF) [file pmed.1000442.s007.pdf]

**Table S3.** The relationship between age group, baseline neutralization titre and seroconversion status.

| | | Fourfold rise in titre | |
|---|---|---|---|
| **Age group (years)** | **Baseline titre** | **Yes** | **No** |
| 3 – 19 | Less than 1:40 | 64 | 43 |
| | 1:40 or greater | 4 | 1 |
| 20 – 39 | Less than 1:40 | 126 | 13 |
| | 1:40 or greater | 7 | 0 |
| 40 – 59 | Less than 1:40 | 349 | 19 |
| | 1:40 or greater | 12 | 1 |
| 60 or older | Less than 1:40 | 122 | 1 |
| | 1:40 or greater | 8 | 0 |
