# Supplementary material for: Epidemiological Characteristics of 2009 (H1N1) Pandemic Influenza Based on Paired Sera from a Longitudinal Community Cohort Study
Source: PLoS Med. 2011 Jun 21;8(6):e1000442. doi: 10.1371/journal.pmed.1000442 (PMC3119689; doi:10.1371/journal.pmed.1000442)
Supplement: Table S4 — Dictionary of field names for Dataset S1 (data from serological study). (0.06 MB PDF) [file pmed.1000442.s008.pdf]

Table S4. Dictionary of field names for Dataset S1 (data from serological study).

Field name	Type	Description and notes
id	String	Individual identifier
gender	String	Either "Male" or "Female"
age	Float	Age on the day of the very first study sample
date.of.baseline	Int	Date of the first blood sample in days since 1st January 1960
date.of.followup	Int	Date of the second blood sample in days since 1st January 1960
child.present	Int	Equal to 1 when a child is present and 2 when they are not present
district	String	Equal to one of: "HK Island", Hong Kong island; "KLN East", Kowloon East; "KLN west", Kowloon west; "NT East", New Territories east; and "NT West", New Territories west
vaccine.0809	String	Self-reported vaccine status for the 2008/2009 seasonal influenza vaccine. Equal to either "Yes" or "No"
recruit.source	Int	Source of recruitment. Equal to 1 for direct recruitment and equal to 2 for recruitment via the parallel telephone survey (see main text)
low.pre.titre	Bool	TRUE if baseline titre was less than 1:40, FALSE otherwise
fourfold.rise	Int	Main outcome measure: equal to 1 if serological testing revealed a fourfold rise or greater in neutralization titres and equal to 0 otherwise
