# Supplementary material for: Epidemiological Characteristics of 2009 (H1N1) Pandemic Influenza Based on Paired Sera from a Longitudinal Community Cohort Study
Source: PLoS Med. 2011 Jun 21;8(6):e1000442. doi: 10.1371/journal.pmed.1000442 (PMC3119689; doi:10.1371/journal.pmed.1000442)
Supplement: Text S1 — A statistical model for severity (0.18 MB PDF) [file pmed.1000442.s009.pdf]

Text S1: A statistical model for severity

Citations are indexed for the reference section in this document.

We defined there to be n participants in the serological survey in a given age group. The vector **b**, with members $b_1 ... b_i ... b_n$ denoted the week in which baseline bloods were taken from each ith member of study. Similarly, the vector **f** denoted the week of follow-up. The vector **x** with members $x_1 \ldots x_i \ldots x_n$ denoted the results of serological assays (see Methods). If the ith individual showed a fourfold rise in titre between baseline and followup, then x_i=1. Otherwise x_i=0.

We defined vector $\boldsymbol{y}$ with members $y_1 \ldots y_j \ldots y_w$ to be the weekly case series of individuals of a given severity level (by onset time) for the whole period for which severe case data are available, i.e. from week 1 to week w the last week for which data were available. Note that the timing of the severe cases is determined by onset, not by admission times or time of death.

We then defined a simple statistical model in which the number of severe cases was used to predict infection. The fundamental assumption of the model was that the severe case data were a sub-sample of the total number of infections that were occurring in the wider community. The size of the age group in the wider population was defined to be N. We defined p as the main parameter of interest, the probability that an infection resulted in a severe case. Therefore, our estimate of the total number of infections with onset in week k was equal to y_k / p. However, many individuals would not have had onsets because they were infected asymptomatically. Therefore, one can think of this as the total number of people who reached the "onset-point" of the natural history of infection in a given week.

We assumed that, for the period of our study, titres against H1N1pdm rose monotonically after onset and remained high until follow-up. However, we did not assume that titres rose immediately. Therefore, we defined the vector **q** with two members q_1 and q_2. We assumed that if an individual had onset (or passed equivalent time of onset) in the same week that a sample was taken, there was zero probability that we would observe a fourfold rise in antibody titre. If onset occurred in the week before the sample, we assumed that we would observe a fourfold rise with probability q_1. If onset occurred two weeks before sampling, we would observe this with

probability q_2. If onset occurred three or more weeks before a sample, we assumed we would definitely observe a titre rise.

Although not directly comparable, we draw on data on confirmed H1N1pdm cases from Figure 2 of Ref [1] to infer antibody kinetic values for $\boldsymbol{q}$. The proportion of individuals with titres greater than or equal to 1:32 rose by 77.5% from 11.6% in the same week as onset of symptoms to 89.1% in the fourth week after symptoms. By the second week, the proportion with titres greater than or equal to 1:32 had risen to 42.9%, or 40.4% of the total 77.5% observed rise. Hence, we took q_1 = 40.4%. Similarly, by the third week after symptoms, the proportion with titres greater than or equal to 1:32 had risen to 84.6%. Therefore, we took q_2 = 94.2%.

We defined r, the likelihood that the ith individual was positive, to be conditional on: the probability p of a severe outcome per infection; the rates that antibodies rise after infection $\mathbf{q}$; the weekly series of severe case incidence by onset $\boldsymbol{y}$; the week of baseline sample b_i; the week of follow-up sample f_i; and the number of people in the age group in the wider population N. Effectively, this formula sums all the severe cases in the population between the baseline and follow-up (with adjustment for rising titres) and multiples the total by p to produce a simple statistical model of infection for participants of the serosurvey,

$$r(i;p,\mathbf{q},\mathbf{y},b_i,f_i,N) = \frac{1}{pN}\sum_{j=1}^{j=w} y_j h(j,b_i,f_i,\mathbf{q}),$$

where,

$$h(j,b_i,f_i,\mathbf{q}) = \begin{cases} 1-q_2, & \text{if } b_i - j = 2 \\ 1-q_1, & \text{if } b_i - j = 1 \\ 1, & \text{if } j \geq b_i \text{ and } j < f_i - 2 \\ q_2, & \text{if } f_i - j = 2 \\ q_1, & \text{if } f_i - j = 1 \\ 0, & \text{otherwise} \end{cases}.$$

This allowed us to calculate the overall likelihood L of the cohort serology results $\boldsymbol{x}$ by taking the generalized product of the individual probabilities,

$$L(\mathbf{x};p,\mathbf{q},\mathbf{y},b_i,f_i,N) = \prod_{i=1}^{i=n} v(x_i;p,\mathbf{q},\mathbf{y},b_i,f_i,N),$$

where,

$$v(x_i; p, \mathbf{q}, \mathbf{y}, b_i, f_i, N) = \begin{cases} r(i; p, \mathbf{q}, \mathbf{y}, b_i, f_i, N) & \text{if } x_i = 1 \\ 1 - r(i; p, \mathbf{q}, \mathbf{y}, b_i, f_i, N) & \text{otherwise} \end{cases}.$$

Because each age group and case series of severe outcomes are independent, we needed only to find the value of a single parameter *p* for which the likelihood was maximized. This was achieved using the golden section search and successive parabolic interpolation as implemented in the function *optimize* in the *base* package of the R statistical framework 2.11.0.

Reference

1. Miller E, Hoschler K, Hardelid P, Stanford E, Andrews N, et al. (2010) Incidence of 2009 pandemic influenza A H1N1 infection in England: a cross-sectional serological study. Lancet.
